# Antibacterial activity mechanism of coptisine against *Pasteurella multocida*

**DOI:** 10.3389/fcimb.2023.1207855

**Published:** 2023-07-12

**Authors:** Rui Zhang, Shuo Tian, Tengfei Zhang, Wenting Zhang, Qin Lu, Qiao Hu, Huabin Shao, Yunqing Guo, Qingping Luo

**Affiliations:** ^1^ Key Laboratory of Prevention and Control Agents for Animal Bacteriosis (Ministry of Agriculture and Rural Affairs), Hubei Provincial Key Laboratory of Animal Pathogenic Microbiology, Institute of Animal Husbandry and Veterinary, Hubei Academy of Agricultural Sciences, Wuhan, China; ^2^ Hubei Hongshan Laboratory, Wuhan, China

**Keywords:** coptisine, *Pasteurella multocida*, antibacterial activity, molecular mechanisms, RNA sequencing

## Abstract

**Objective:**

*Pasteurella multocida* is a widespread zoonotic pathogen that causes severe damage to the poultry industry. This study focused on the antibacterial effects and mechanism of action of coptisine against *P. multocida*.

**Methods:**

The minimum inhibitory concentration and half maximal inhibitory concentration of coptisine against *P. multocida* was measured. Additionally, the effect of coptisine on growth, cell wall, activity of respiratory enzymes, soluble protein content and DNA synthesis were also analyzed. Finally, the effect of coptisine on gene transcription was determined using RNA sequencing.

**Results:**

We demonstrated that coptisine has a strong antibacterial effect against *P. multocida*, with a minimum inhibitory concentration of 0.125 mg/mL. Moreover, the measurement of the half maximal inhibitory concentration confirmed that coptisine was safe for the pathogen. The growth curve showed that coptisine inhibited bacterial growth. Measurement of alkaline phosphatase activity in the culture solution showed that coptisine affected cell wall permeability. Transmission electron microscopy revealed that coptisine chloride destroyed the cell structure. In addition, coptisine blocked the respiratory system, as measured by the levels of critical enzymes of the tricarboxylic acid cycle and glycolysis, succinate dehydrogenase and lactate dehydrogenase, respectively. Similarly, coptisine inhibited the synthesis of soluble proteins and genomic DNA. The KEGG pathway analysis of the differentially expressed genes showed that they were associated with cellular, respiratory, and amino acid metabolism, which were downregulated after coptisine treatment. Additionally, genes related to RNA degradation and the aminoacyl-tRNA pathway were upregulated.

**Conclusion:**

In this study, we demonstrated that coptisine exerts an antibacterial effect on *P. multocida*. These findings suggest that coptisine has a multifaceted impact on various pathways, resulting in the inhibition of *P. multocida*. Thus, coptisine is a potential alternative to antibiotics for the treatment of *P. multocida* infections in a clinical setting.

## 1 Introduction

*Pasteurella multocida* is a non-motile, non-spore-forming, gram-negative bacterium with no flagella (Peng et al., 2019). *P. multocida* is a widespread zoonotic pathogen with a multitude of hosts, including birds, cattle, swine, and humans (Harper et al., 2006). These bacteria can cause pneumonia, hemorrhagic septicemia, and fowl rhinitis, leading to high morbidity, mortality, and significant economic losses on large farms worldwide (Hurtado et al., 2018). *P. multocida* can be classified into many serotypes using different methods, such as those based on its outer capsule and lipopolysaccharides (Smith et al., 2021). Limited cross-protection among serotypes makes prevention more difficult. Therefore, antimicrobial therapy is widely used in clinical trials to treat infections caused by *P. multocida*.

Antimicrobial therapy has many drawbacks, such as multidrug resistance and food safety issues. The excessive consumption of antimicrobials by animals accelerates multidrug resistance in bacteria. *P. multocida* strains isolated from cattle show increasing resistance to tetracycline, tilmicosin, flumequine, and fluoroquinolones (Bourély et al., 2019). *P. multocida* strains isolated from swine were tested for resistance to lincomycin (Cuevas et al., 2020). Additionally, *P. multocida* isolated from different hosts were all multi-drug resistant to various antibiotics. *P. multocida* isolated from different regions also had multi-drug resistance. For instance, among the 20 strains of *P. multocida* in Australia, 70% were resistant to tetracycline. Two strains isolated from two farms in the UK were both resistant to florfenicol (Chen et al., 2022). Of particular concern is that the antibiotic resistance of bacteria isolated from humans is similar to that of bacteria isolated from food-producing animals (European Centre for Disease Prevention and Control (ECDC) et al., 2021). The presence of drug-resistant bacteria poses a significant challenge to the treatment of bacterial infections.

With the growing problem of antibiotic resistance, it is necessary to exploit new antibiotic alternatives. Traditional medicines and natural products have incomparable advantages in treating bacterial infections (Yuan et al., 2016). Traditional Chinese medicine is one of the most important approaches with a curative effect on viral and bacterial infectious diseases (Ma et al., 2019). Traditional Chinese medicine has the advantages of less toxic side effects and less susceptibility to drug resistance (Wiseman, 2002). *Coptis chinensis* is one of the most common constituents of traditional Chinese herbal medicines with large-scale cultivation in China. Alkaloids, the main active component of *C. chinensis*, have anticancer, anti-inflammatory, and antibacterial properties (Wang et al., 2019). Epiberberine, coptisine, palmatine, and berberine are the major alkaloids of *C. chinensis*, as confirmed by the Chinese Pharmacopoeia (Chinese Pharmacopoeia Commission. Chinese Pharmacopoeia, 2020). Berberine is the most abundant alkaloid in *C. chinensis*, possessing multiple pharmacological effects, and is widely used in the clinical setting (Wang et al., 2017). Coptisine is the second most abundant alkaloid in this plant. Like berberine, coptisine also exhibits numerous pharmacological and biological properties (Mi et al., 2015). The antibacterial effects of coptisine against multiple pathogenic bacteria have been reported previously. For example, coptisine inhibits *Helicobacter pylori via* the inactivation of bacterial urease through direct binding to a sulfhydryl group in the enzyme’s active site (Li et al., 2018). Coptisine also exerts antimicrobial activity against *Escherichia coli*, and the methylenedioxy groups at C2 and C3 on its phenyl ring contribute to this activity (Yan et al., 2008). Furthermore, coptisine has been found to decrease the adhesion of *Staphylococcus aureus* to human lung epithelial cells (Wang et al., 2018). Thus, coptisine can act as a potential antibacterial agent in the clinical setting, promoting the utilization of high-quality *C. chinensis.*

Our preliminary study showed that *C. chinensis* had good antibacterial activity against *P. multocida*. However, the antibacterial effects and mechanisms of action of coptisine against *P. multocida* remain unknown. Thus, in the present study, we investigated the antimicrobial activity of coptisine against *P. multocida*. Genes regulated by coptisine were identified using RNA sequencing (RNA-seq). The elucidation of the mechanism and target genes will provide a theoretical basis for antibiotic substitution in *P. multocida*.

## 2 Materials and methods

### 2.1 Bacterial strains, media, and growth conditions

The *P. multocida* strain used in this study was C48-1, which was purchased from the China General Microbiological Culture Collection Center (CGMCC). *P. multocida* was cultured at 37°C in Difco tryptic soy broth (TSB) or tryptic soy agar (TSA) (BD, Franklin Lakes, NJ, USA).

### 2.2 Chemicals and reagents

Coptisine (purity > 98%) and sodium dodecyl-sulfate polyacrylamide gel electrophoresis (SDS-PAGE) kits were purchased from Solarbio (Beijing, China). Alkaline phosphatase (AKP), succinate dehydrogenase (SDH), and lactate dehydrogenase (LDH) activity assay kits were obtained from the Nanjing Jiancheng Bioengineering Institute (Jiancheng, Nanjing, China). The Bradford protein concentration assay kit was purchased from Beyotime Biotechnology (Shanghai, China).

### 2.3 Minimum inhibitory concentration and half maximal inhibitory concentration assay

The minimum inhibitory concentration (MIC) was measured using the dilution method (Kowalska-Krochmal and Dudek-Wicher, 2021). The initial concentration of coptisine in the first well was 2 mg/mL, and the solution was diluted 2-fold. Finally, 100 μL of bacterial suspension (McFarland standard 0.25) was added. After culturing at 37°C for 24 h, the MIC of coptisine was identified as the well with no bacterial growth.

The half maximal inhibitory concentration (IC_50_) was measured as previously described (Chen et al., 2021). A CCK-8 kit (Yeasen, Shanghai, China) was used to evaluate the effect of coptisine on the viability of DF-1 cells. DF-1 cells, in 100 μL of culture medium, were added at a density of 10^4^ cells/mL to 96-well plates. The cells were treated with coptisine at various concentrations for 24 h. Subsequently, the IC_50_ was estimated according to the manufacturer’s instructions. The experiments were performed in triplicate.

### 2.4 Antibacterial kinetic curve determination

A growth curve was constructed using the viable cell count method. Briefly, independent bacterial colonies were inoculated in TSB and cultured overnight with shaking. The resulting bacterial culture was then transferred to fresh TSB medium containing different concentrations of coptisine (0, 0.5 MIC, MIC, and 2 MIC) at a ratio of 1:1000 (v/v) and grown at 37°C with shaking at 200 rpm. Colony-forming units (CFUs) were measured by agar pouring inoculation, and this test was done at 2-h intervals for a total of 18 h.

### 2.5 Cell membrane integrity of *P. multocida*

The morphological characteristics of *P. multocida* cell membranes were observed using transmission electron microscopy (TEM). *P. multocida* cultured at an initial concentration of 10^7^ CFU/mL was exposed to the MIC concentration of coptisine, and the mixtures were then incubated at 37°C with shaking at 200 rpm for 4 and 8 h, respectively. Cultures without coptisine were used as controls. Cells were collected, dehydrated, embedded, and stained as previously described (Feng et al., 2016). The morphologies of the prepared samples were observed using TEM.

### 2.6 Cell permeability of *P. multocida*

A suspension of *P. multocida*, cultured to the mid-exponential phase, was transferred to fresh TSB containing the MIC of coptisine. The initial concentration of the medium was 10^7^ CFU/mL. The mixtures were incubated at 37°C with shaking at 200 rpm, and cultures without coptisine were used as controls. Samples were collected from each tube at 0, 2, 4, 6, 8, 10, and 12 h. The activity of AKP was measured using an AKP activity assay kit.

### 2.7 Activities of respiratory enzymes

*P. multocida* was cultured in TSB until it reached the mid-exponential phase. Then, the culture was incubated at 37°C with shaking at 200 rpm for 10 h. Three separate experiments were performed for each group. The suspension was centrifuged at 5000 rpm for 10 min, and the sediment of the bacterial sludge was washed three times with a 0.1 mol/L Tris-HCl buffer. Then, an equal volume of lysozyme solution was added to the bacterial sludge, incubated at 37°C for 20 min, and then immediately placed in an ice bath. The solution was mixed with 1 mL Tris-SDS buffer and centrifuged at 10,000 rpm for 15 min at 4°C. The SDH and LDH activities in the supernatants were determined according to the manufacturer’s instructions.

### 2.8 Determination of soluble protein content

The *P. multocida* strain was cultured in TSB until it reached the mid-exponential phase. The culture was transferred to fresh TSB containing different concentrations of coptisine (0, 0.5 MIC, MIC, 2 MIC) at a ratio of 1:1000 (v/v). The culture was incubated at 37°C with shaking at 200 rpm for 10 h. A medium without coptisine was used as a control. The number of cells was adjusted to the same value, and the cells were collected by centrifugation. The precipitate was suspended with loading buffer and boiled at 100°C for 10 min. The supernatant was prepared by centrifugation at 4000 rpm for 10 min at 4°C. The supernatant was resolved with SDS-PAGE. The gel was stained with 0.05% Coomassie Brilliant Blue R-250 and de-stained to obtain soluble protein bands. The protein bands were quantified using the Gel-Pro Analyzer 4 software.

### 2.9 Determination of genomic DNA content

The content of genomic DNA was determined using gel electrophoresis and DAPI staining methods. *P. multocida* was cultured until the mid-exponential phase. The culture was adjusted to a concentration of 1×10^6^ CFU/mL using TSB. Following the addition of the MIC of coptisine, the culture was then incubated at 37°C for 30, 60, 90, and 120 min. Sterilized water was used as the control. The number of cells was adjusted to the same value, and the cells were collected by centrifugation. Genomic DNA was extracted using a DNA extraction kit (TianGen, Beijing, China) and analyzed by 1% agarose gel electrophoresis. The optical densities of the bands were analyzed using Gel-Pro Analyzer 4 software.

The DAPI assay was performed as described previously (Liu et al., 2022). First, *P. multocida* culture was added to the TSB medium containing the MIC of coptisine at a final concentration of 1×10^6^ CFU/mL. The cultures were kept at 37°C with shaking at 200 rpm for 10 h. The number of cells was adjusted to the same value, and they were suspended in the same volume. Subsequently, 1 μg/mL DAPI was added to the 1 mL suspension and incubated in the dark at 25°C for 1 h. The intensity of the blue DNA fluorescence was then observed under a fluorescence microscope.

### 2.10 RNA-seq of genes regulated by coptisine

*P. multocida* was cultured in TSB until it reached the mid-exponential phase. The culture was then transferred to fresh TSB at 1:100 (v/v). The culture was incubated at 37°C with shaking at 200 rpm. A 2 MIC concentration of coptisine was added to the culture at an OD600 of 0.6. The cultures were maintained for 2 h under similar conditions. A medium without coptisine was used as a control. Total RNA was extracted using an RNA Extraction Kit (Omega, Norcross, GA, USA). The total RNA was treated with RNase-free DNase I (Promega, Wisconsin, USA) at 37°C for 30 min to obtain the purified RNA. Purified RNA was sent to Shanghai Majorbio Biopharm Technology Co., Ltd. (Shanghai, China) for RNA-seq. All RNA samples were analyzed in triplicate.

Differentially expressed genes were verified using reverse transcription quantitative real-time PCR (RT-qPCR). Primers were designed using the Primer 6.0 software; the sequence of primers is shown in the **Supplementary Material**. RNA was extracted as previously described. RNA was reverse transcribed to cDNA using the PrimerScript RT Reagent Kit with a gDNA Eraser (Takara, Dalian, China). RT-qPCR was performed using SYBR Premix (Vazyme, Nanjing, China). The 16S rRNA gene was used as a reference gene. Relative quantification of gene expression was calculated using the 2^-△△Ct^ method.

### 2.11 Statistical analysis

All experiments were performed in triplicate. Student’s *t*-test was used to determine whether there was a significant difference (*P* < 0.05) using Prism software (GraphPad Software, San Diego, CA, USA).

## 3 Results

### 3.1 Effect of coptisine on the growth of *P. multocida*

**Figure 1:**
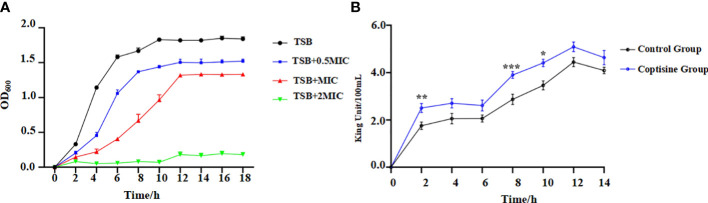
Antibacterial kinetic curves of coptisine against *P. multocida* and determination of extracellular alkaline phosphatase (AKP) activities. **(A)** Antibacterial kinetic curves of coptisine against *P. multocida* with different concentrations of coptisine. **(B)** Extracellular AKP activities. *:0.01<P<0.05; **:0.001<P<0.01; ***:P<0.001.

The MIC of coptisine against C48-1 was 0.125 mg/mL, whereas methyl alcohol (12.5%) had no effect on the growth of *P. multocida*. The IC_50_ of coptisine on DF-1 cells was found to be greater than 2 mg/mL, which is the concentration of its mother liquor, thus indicating its safeness to the organism. The antibacterial kinetic curves are shown in **Figure 1A**. The strain grown in the medium containing coptisine showed a slower growth rate than that grown in the medium without coptisine. The 2 MIC concentration of coptisine completely inhibited strain growth. This indicates that coptisine has a strong inhibitory activity on *P. multocida*, which occurred in a concentration-dependent manner.

### 3.2 Effect of coptisine on the cell wall of *P. multocida*

Cell membrane permeability was assessed by measuring extracellular AKP activity (Liu et al., 2022). The extracellular activity of AKP in the coptisine treatment group was significantly higher than that in the control group (**Figure 1B**). This result reveals that AKP leaked into the extracellular space, indicating that coptisine increases the normal permeability of bacterial cell membranes.

**Figure 2:**
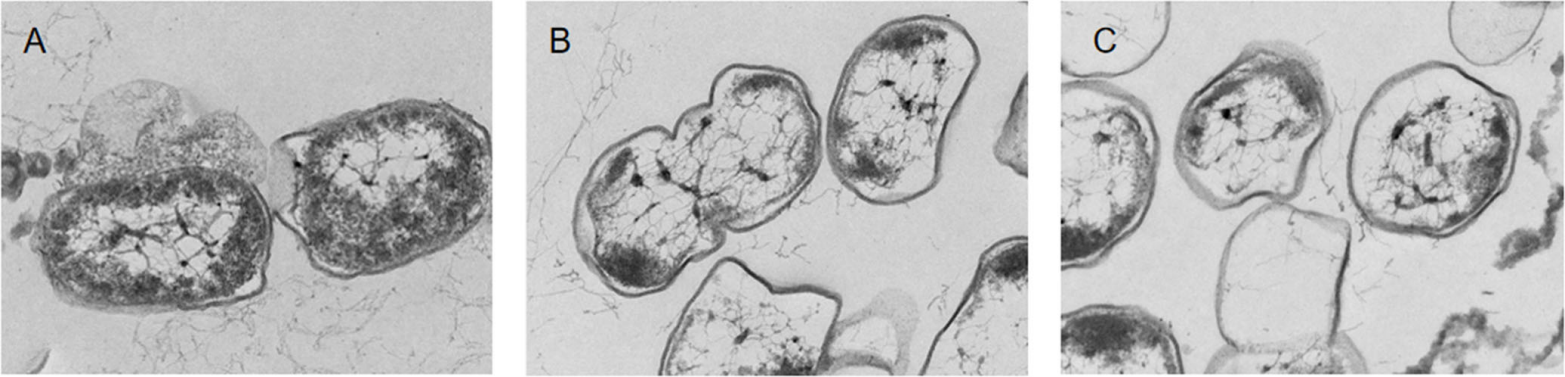
Transmission electron microscopy images of coptisine-treated and untreated *P. multocida* cells. **(A)** Untreated cells. **(B)** Cells treated with the minimum inhibitory concentration (MIC) of coptisine for 4 h. **(C)** Cells treated with the MIC of coptisine for 8 h.

The integrity of the cell membrane structure was observed using TEM. The TEM image of the untreated cells was smooth and complete, and the chromatin was homogeneously distributed in the center of the cell (**Figure 2A**). After coptisine treatment for 4 h, membrane integrity and cytoplasmic distribution were slightly disrupted (**Figure 2B**). After coptisine treatment for 8 h, the cell damage became more severe, and the cells exhibited morphological deformations and cytoplasmic vacuolation (**Figure 2C**). This result shows that coptisine destroys the structure of *P. multocida*.

### 3.3 Effect of coptisine on the activity of respiratory enzymes of *P. multocida*

The SDH activity of cells treated with coptisine at the MIC was 5.80 ± 0.74 U/mg protein, which was significantly lower than that of untreated cells (31.53 ± 1.27 U/mg protein; *P* < 0.05). In addition, LDH activity significantly decreased (*P* < 0.05). The values of LDH activity of cells treated with and without coptisine were 6.53 ± 0.36 and 3.51 ± 0.42 U/mg protein, respectively. After treatment with coptisine, the SDH and LDH levels decreased by 81.6% and 46.4%, respectively. This reveals that coptisine inhibits the activity of SDH and LDH, both of which are involved in the respiratory system of *P. multocida*.

### 3.4 Effect of coptisine on the soluble protein content of *P. multocida*

**Figure 3:**
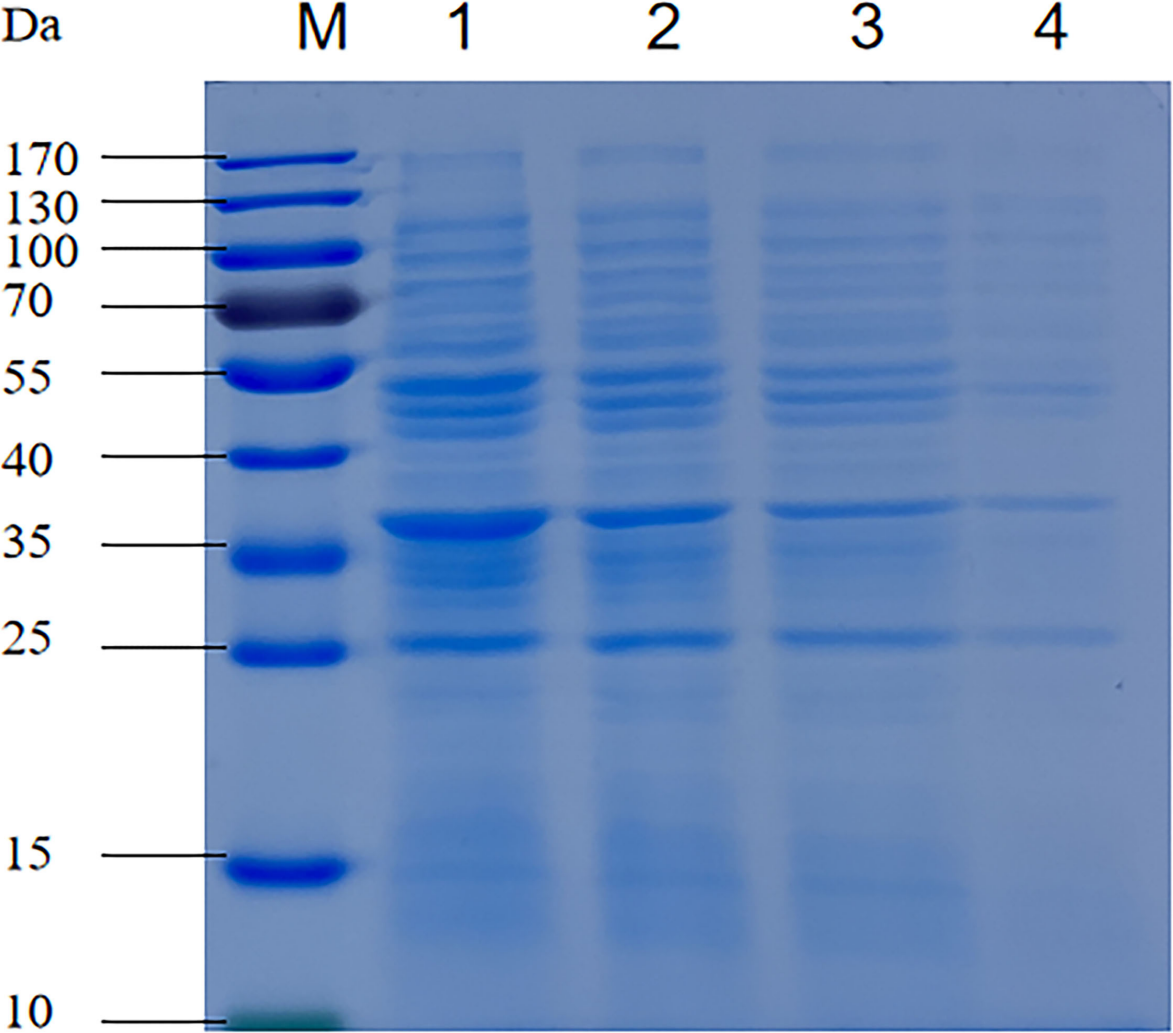
Sodium dodecyl-sulfate polyacrylamide gel electrophoresis profiles of the soluble protein content of coptisine-treated and untreated *P. multocida* cells. Lane 1, untreated cells; lane 2, cells treated with half of the minimum inhibitory concentration (MIC) of coptisine; lane 3, cells treated with the MIC of coptisine; lane 4, cells treated with twice the MIC of coptisine.

The effects of coptisine on the soluble protein content of *P. multocida* are shown in **Figure 3**. The protein bands of cells treated with coptisine at the MIC were significantly lower than those in the untreated group, as depicted in **Figure 3**. Furthermore, as the dose of coptisine increased, the protein bands progressively decreased. The amount of soluble protein in the 0.5 MIC, MIC, and 2 MIC treatment groups was reduced by 4.5, 14.5, and 28.1%, respectively, when compared with the untreated group. The results show that coptisine inhibits soluble protein synthesis in *P. multocida*.

### 3.5 Effect of coptisine on DNA synthesis in *P. multocida*

**Figure 4:**
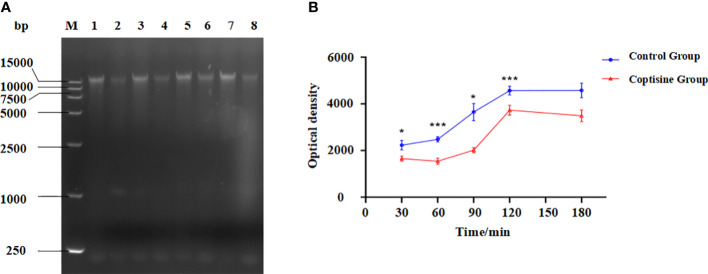
Genomic DNA content of coptisine-treated and untreated *P. multocida* cells. **(A)** Agarose gel electrophoresis map of genomic DNA content. M: DL 15000 DNA Marker; Lane 1, 3, 5, 7: untreated cells incubated with sterilized water for 30, 60, 90, and 120 min, respectively; Lane 2, 4, 6, 8: *P. multocida* cells incubated with the minimum inhibitory concentration of coptisine for 30, 60, 90, and 120 min, respectively. **(B)** The optical density of the genomic DNA content of coptisine-treated and untreated cells. *:0.01<P<0.05; ***:P<0.001.

**Figure 5:**
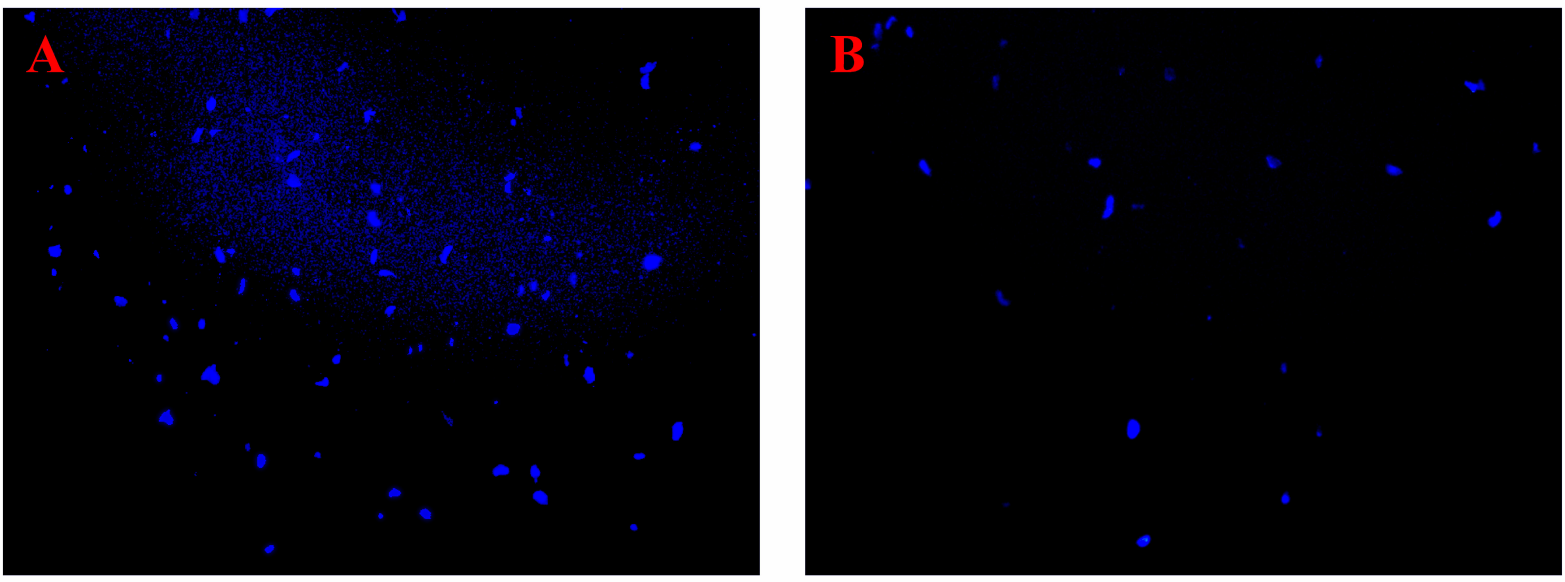
DAPI fluorescence of coptisine-treated and untreated *P. multocida* cells. **(A)** Cells treated with coptisine. **(B)** Untreated cells.

The genomic DNA content of *P. multocida* cells is shown in **Figure 4**. The bands corresponding to genomic DNA content gradually increased over time. The bands in the control group were deeper than those in the coptisine-treated group (**Figure 4A**). The gray value was measured using gel analysis software (**Figure 4B**). The genomic DNA content of *P. multocida* cells treated with coptisine at MIC was significantly lower than that of the control group. This indicates that coptisine continuously inhibits the synthesis of bacterial genomic DNA. The DAPI assay results showed that the fluorescence of the coptisine group was significantly less than that of the control group (**Figure 5**). Since the number of cells was adjusted to be the same, the inhibition of bacterial genomic DNA synthesis by coptisine is implied, which is consistent with the results obtained from the measurement of genomic DNA content.

### 3.6 Transcriptional response in the presence of coptisine

**Figure 6:**
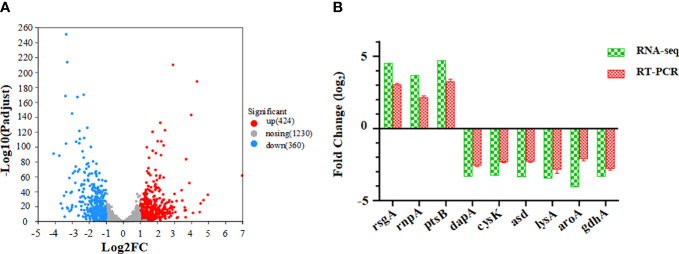
Differentially expressed genes between coptisine-treated and untreated cells. **(A)** Volcano plot showing the differentially expressed genes between coptisine-treated and untreated cells. **(B)** Comparison of the fold change of some differently expressed genes between RNA sequencing and reverse transcription quantitative real-time PCR methods.

Gene expression was compared between cells grown under normal conditions and those cultured in the presence of coptisine. The differentially expressed genes between samples were assigned according to the |log2 ratio| >1 and *P* ≤ 0.05 (Love et al., 2014). In total, 424 genes were upregulated, and 360 were downregulated, as shown in **Figure 6A**. The trends in RT-qPCR were consistent with the results observed in the RNA-seq analysis, as illustrated in **Figure 6B**.

**Figure 7:**
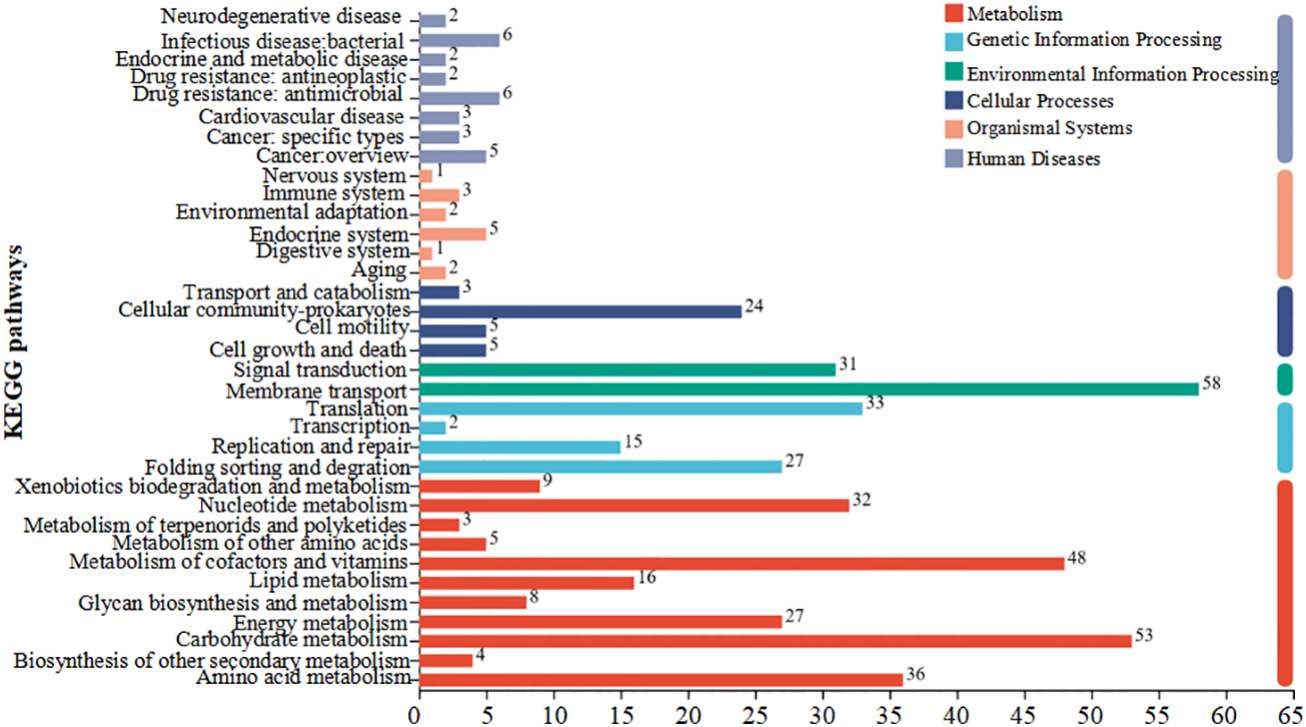
KEGG pathway analysis of the differentially expressed genes regulated by coptisine.

**Figure 8:**
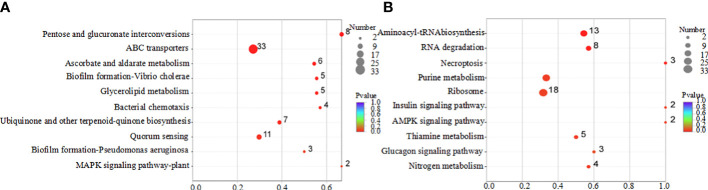
KEGG enrichment analysis of differentially expressed genes regulated by coptisine. **(A)** Downregulated genes. **(B)** Upregulated genes.

According to the KEGG pathway classification, differentially expressed genes were primarily involved in amino acid metabolism, genetic information processing, and cellular processes (**Figure 7**). The results of the pathway enrichment analysis of the differentially expressed genes are shown in **Figure 8**. Downregulated genes were primarily involved in ABC transporters, quorum, and amino acid metabolism (**Figure 8A**). The upregulated genes were mainly involved in ribosome and aminoacyl-tRNA biosynthesis and RNA degradation (**Figure 8B**).

## 4 Discussion

Traditional Chinese medicine can offer several advantages, such as less toxic bioactive compounds, biodegradability, and a broad spectrum of secondary metabolites, which make it a popular alternative source in antibiotic preparation (Abdallah et al., 2019). Antimicrobial activities of traditional Chinese medicine include inhibition of bacterial cell growth, biofilm formation, and disruption of bacterial quorum-sensing (Wang et al., 2011; Yang et al., 2020; Yang et al., 2022). This study aimed to explore the antimicrobial activity of coptisine against *P. multocida* and its mode of action.

It was demonstrated that coptisine exerted antibiotic activity against *P. multocida*, with an MIC of 0.125 mg/mL. Moreover, the IC_50_ of coptisine on DF-1 cells was much larger than its MIC, indicating that coptisine was safe and effective. TEM revealed that coptisine damaged the cell membrane integrity of *P. multocida*. In addition, AKP, located in the bacterial cell wall, was released into the extracellular space, consistent with an increase in cell membrane permeability (Zhang et al., 2016). Changes in cell membrane permeability also lead to the leakage of nucleic acids, proteins, and small molecular substances, which accelerates bacterial death (Kohanski et al., 2010). Thus, coptisine-induced damage to the bacterial membrane may be a key feature of its antibacterial activity.

Respiratory metabolism is known to be associated with bacterial activity. The tricarboxylic acid (TCA) cycle is central to energy production and biosynthetic precursor synthesis in bacteria (Kwong et al., 2017). LDH and SDH are enzymes involved in cellular metabolism. LDH is involved in the conversion of pyruvate to lactate during anaerobic glycolysis, while SDH is a key enzyme in the TCA cycle involved in the conversion of succinate to fumarate (Pan et al., 2022). It was demonstrated that the SDH and LDH activity of *P. multocida* was significantly decreased after coptisine treatment. Genes related to the TCA cycle and glycolysis, including *fumC, araD, lyx, pgi*, and *sucD*, were significantly downregulated. This reveals an antibacterial action mechanism of coptisine that affects the energy metabolism of *P. multocida*.

In the present study, coptisine was found to inhibit multiple pathways associated with cellular metabolism. Specifically, the treatment with coptisine resulted in a significant reduction in both DNA and soluble protein content. Transcription analysis revealed a significant downregulation of key genes involved in purine pyrimidine anabolism, including *cyaA, gmk, mazG, nrdD, ribD*, and *cmkA*. It was also reported that coptisine perturbed the ctDNA structure and acted as an inhibitor, thereby decreasing urea synthesis in the liver and urea-N emission in the urine (Ran Mi et al., 2015). Furthermore, genes involved in amino acid synthesis, such as *hisB*, *lysA*, and *argD*, were also significantly downregulated. The RNA-seq results were consistent with those of the phenotypic analysis of DNA and soluble protein synthesis. Thus, coptisine inhibits the synthesis of numerous proteins. Previously, it was shown to inhibit the activity of ruminal bacterial urease activity (He et al., 2022), while also acting as an inhibitor of the pathway of ONOO^–^mediated protein tyrosine nitration (Choi et al., 2015).

Moreover, among the downregulated genes screened by RNA-Seq, the KEGG pathway enrichment emphasized amino acid metabolism, DNA synthesis, TCA, iron utilization, and glycolysis. This was consistent with changes in DNA content, soluble protein release, and the activities of SDH and LDH. It has been reported that coptisine has anti-cancer properties that involve its effect on the glucose metabolism of cancer cells (Lv et al., 2020). Coptisine also plays a role in ferrous ion chelating activity (Jang et al., 2009). *H. pylori* was also effectively inhibited by coptisine through a reduction in its urease activity (Li et al., 2018). Conversely, the upregulated genes were primarily involved in RNA degradation, aminoacyl-tRNA biosynthesis, and cytochrome C-type proteins. Aminoacyl-tRNA plays a pivotal role in amino acid transport and is important for bacterial survival (Lv and Zhu, 2012). Cytochrome C is an essential intermediate protein in ATP production and a potential antibacterial target (Pessoa, 2021).

The current study focused on the phenotype and related genes regulated by coptisine. As coptisine has multiple functions, it has attracted much research interest. Coptisine can inhibit the NF-κB, NLRP3, and p38 MAPK signaling pathways (Luo et al., 2018; Wu et al., 2019). Coptisine can also bind to the virulence factor SrtB of *S. aureus*, thereby reducing its infection (Wang et al., 2018). In addition, coptisine exerts a hypolipidemic effect by reducing mRNA levels of p65, VCAM-1, ICAM-1, and IL-6/1β in the aorta and liver (Feng et al., 2017). Berberine exhibits antibacterial activity by binding to nucleic acids, inhibiting the activity of different enzymes, and causing morphological changes (Aksoy et al., 2020). It has also been shown that chloroform extract from black pepper possesses antibacterial properties, which involve inhibition of bacterial metabolic pathways [33]. This illustrates that the antibacterial activity of traditional Chinese medicines occurs through multiple mechanisms. The results of this study also indicate that coptisine has a variety of pharmacological effects through different signaling pathways.

In summary, coptisine exerts an antibacterial effect on *P. multocida*. The associated mechanisms involve the inhibition of cellular metabolism, including amino acid synthesis and respiratory metabolism, damage to cell structures, and an increase in cell wall permeability. RNA-seq confirmed that coptisine affects the transcription of the genes involved in these processes. In conclusion, this is an application-based study on the metabolism of *P. multocida*, which may be helpful in the treatment of infections caused by this pathogen.

## Data availability statement

The datasets presented in this study can be found in online repositories. The names of the repository/repositories and accession number(s) can be found below: https://www.ncbi.nlm.nih.gov/, PRJNA956698.

## Author contributions

RZ and ST performed the experiments. TZ, WZ, and HS provided guidance during the experimental methods. QH and QLu contributed reagents and analytical tools. YG and QLuo designed the experiments and revised the manuscript. All authors contributed to the article and approved the submitted version.
