# Supplementary material for: Antibacterial activity mechanism of coptisine against Pasteurella multocida
Source: Front Cell Infect Microbiol. 2023 Jul 12;13:1207855. doi: 10.3389/fcimb.2023.1207855 (PMC10369072; doi:10.3389/fcimb.2023.1207855)
Supplement: Supplementary file 1 [file Table_1.doc]

Table S1 The primers of RNA-seq used in this study

| Genes name | Primers (5’–3’) | Length (bp) |
| --- | --- | --- |
| *aorA*-F | AACCTTTGGCGTTGAAGTTG | 130 |
| *aroA*-R | CGCGGCAAGAAAATAAGAAG |
| *hisB*-F | AAGGCGATTTGTGGATTGAC | 136 |
| hisB-R | TTGCATTCGTCCATTGGTAA |
| lysA-F | TGTGGCGAAAACAGAAACAG | 112 |
| lysA-R | CATTTCACCCGCTACCTGAT |
| Asd-F | TCGTGCTTGATCCAGTGAAC | 111 |
| Asd-R | CGCCTAACGCCATTAACATT |
| gdhA-F | ATGACGCAAAGTTCACAACG | 115 |
| gdhA-R | GACCTTCAATCGTGCCGTAT |
| dapA-F | CGAATCCAATCCAATTCCTG | 133 |
| dapA-R | CGCGATTTTTAATGCGTCTT |
| cysK-F | CAAATTGATGTGCTGGTTGC | 121 |
| cysK-R | GTGATTCCGCAGGTTCAACT |
| ptsB-F | ATTATTCCTGCCATCGTTGC | 110 |
| ptsB-R | ATTGCGGGATACAGATCGAC |
| rsgA1-F | CAGCTGCCTTTCTTTTACGC | 119 |
| rsgA1-R | ATTTTTCGGGGCTTCAATTT |
| rnpA-F | GGGTTAACTGTCGCGAAAAA | 127 |
| rnpA-R | TCGCCACAATAACAAAATCG |
